# Fundamental and practical approaches for single-cell ATAC-seq analysis

**DOI:** 10.1007/s42994-022-00082-5

**Published:** 2022-09-27

**Authors:** Peiyu Shi, Yage Nie, Jiawen Yang, Weixing Zhang, Zhongjie Tang, Jin Xu

**Affiliations:** 1 State Key Laboratory of Biocontrol, School of Life Sciences, Sun Yat-Sen University, Guangzhou, 510275 China; 2 Zhongshan School of Medicine, Sun Yat-Sen University, Guangzhou, 510275 China

**Keywords:** Chromatin accessibility, scATAC-seq, Data analysis, Bioinformatic tools

## Abstract

Assays for transposase-accessible chromatin through high-throughput sequencing (ATAC-seq) are effective tools in the study of genome-wide chromatin accessibility landscapes. With the rapid development of single-cell technology, open chromatin regions that play essential roles in epigenetic regulation have been measured at the single-cell level using single-cell ATAC-seq approaches. The application of scATAC-seq has become as popular as that of scRNA-seq. However, owing to the nature of scATAC-seq data, which are sparse and noisy, processing the data requires different methodologies and empirical experience. This review presents a practical guide for processing scATAC-seq data, from quality evaluation to downstream analysis, for various applications. In addition to the epigenomic profiling from scATAC-seq, we also discuss recent studies in which the function of non-coding variants has been investigated based on cell type-specific cis-regulatory elements and how to use the by-product genetic information obtained from scATAC-seq to infer single-cell copy number variants and trace cell lineage. We anticipate that this review will assist researchers in designing and implementing scATAC-seq assays to facilitate research in diverse fields.

## Introduction

In multicellular organisms, cellular heterogeneity is the basis for distinct physiological functions and affects a wide range of biological processes, including developmental plasticity (Chang et al. 2008) and cancer heterogeneity (Dagogo-Jack and Shaw 2018). With the advent of single-cell sequencing technologies, cell-to-cell variations have been characterized at the molecular level (Han et al. 2020). As an example, single-cell RNA-seq, which allows researchers to profile the whole transcriptome of a large number of individual cells, has been applied to explore novel or rare cell populations (Villani et al. 2017) and to uncover the diversity of immune cells in tumors (Zheng et al. 2021). Although the transcriptome information in individual cells has successfully been used to reveal the identity and functions of cells, the underlying mechanisms that regulate cellular diversity are not completely understood and have attracted much attention as a research topic (Han et al. 2020).

Accessibility of chromatin is one of the main epigenetic regulatory layers. It can be measured using various high-throughput sequencing assays, such as DNase I hypersensitive sites sequencing (DNase-seq), transposase-accessible chromatin sequencing (ATAC­seq), micrococcal nuclease sequencing (MNase-seq), and nucleosome occupancy and methylome sequencing (NOMe-seq) (Klemm et al. 2019). Among these methods, ATAC­seq has gained growing popularity owing to its efficiency and sensitivity (Buenrostro et al. 2013).

With the development of single-cell ATAC sequencing (scATAC-seq), the study of chromatin accessibility has been extended to single-cell resolution (Buenrostro et al. 2015). However, the processing of scATAC-seq data, which tends to be sparse and noisy, requires different methodologies (Schep et al. 2017; Fang et al. 2021). The lack of a comprehensive handbook on scATAC-seq data analysis may hinder its further applications. This review provides a brief overview of the general principle and production of single-cell ATAC-seq followed by an explanation of the data analysis process from the general workflow to downstream analysis for different applications. We also discuss key analytical tools for processing the scATAC-seq datasets.

## Sample preparation and quality control for scATAC-seq

**Fig. 1:**
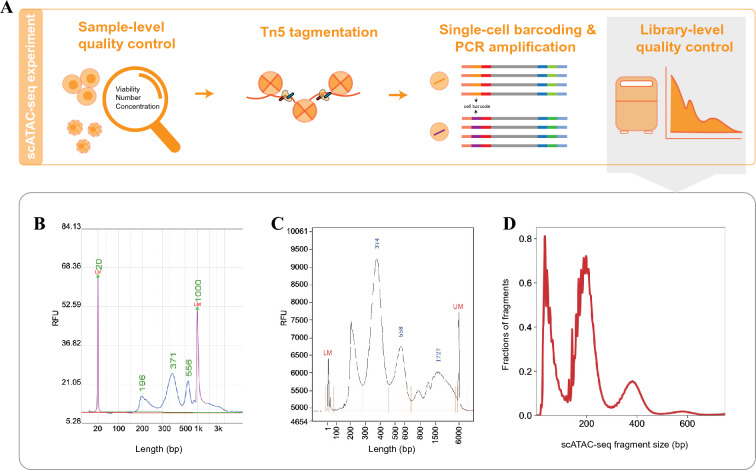
General steps and quality control of a conventional scATAC-seq experiment. **A** Schematic summary of scATAC-seq library generation. **B** Length distribution of library fragment quantified by Qseq. **C** Length distribution of library fragment quantified by Agilent Bioanalyzer 2100. **D** Length distribution of DNA fragment by sequencing

ATAC-seq utilizes a genetically engineered hyperactive Tn5 transposase to insert adaptors into accessible chromatin regions, thereby enabling genome-wide profiling of open chromatin regions by sequencing (Fig. 1A). Following the development of bulk ATAC-seq methods, three different strategies for single-cell ATAC-seq have been developed, including the microfluidics-based method (Buenrostro et al. 2015), the split-and-pool combinatorial cellular indexing method (Cusanovich et al. 2015), and the droplet-based procedure (Satpathy et al. 2019). Initial single-cell ATAC-seq technologies using microfluidics or split-and-pool combinatorial cellular indexing have the disadvantages of low throughput and high costs. Recently, several methods integrated with fluorescence-activated cell sorting (FACS), droplet, and nano-well platforms have been developed by either commercial companies or academic groups. These methods provide high-throughput solutions and are becoming increasingly popular. The experimental details and protocols for each method have been previously reviewed and discussed (Baek and Lee 2020; Preissl et al. 2022). Here we would like to focus on the issues of sample preparation and preservation, which have not been adequately covered in the literature.

Initially, ATAC-seq was developed for fresh cells or cells disassociated from fresh tissues (Buenrostro et al. 2015; Cusanovich et al. 2015). As ATAC-seq quantifies DNA, which is more stable than RNA molecules, it can be further applied to frozen tissues when nuclei could be well isolated (Corces et al. 2017; Cusanovich et al. 2018b). With the optimized ATAC-seq protocol, nuclei are more readily used for scATAC-seq, especially for tissues that are difficult to dissociate into single cells (Rai et al. 2020; Ziffra et al. 2021). Furthermore, Chen et al. demonstrated that cells or nuclei fixed with formaldehyde yield ATAC-seq data similar to fresh cells (Chen et al. 2016), which provides an optimized approach to maintain the integrity of cells or nuclei during single-cell separation in many scATAC-seq methods. In summary, it has been shown in many studies that scATAC-seq can be applied to fresh tissues, as well as frozen or fixed samples, in contrast to scRNA-seq. We highlight the publications that performed scATAC-seq on different tissues with various sample preservation and preparation methods (Table 1).

**Table 1:** Summary of scATAC-seq experiments with different sample preparation and preservation protocols

Sample preservation	Sample preparation	Tissues	Reference
Fresh	Cell	Cell line, PBMC	(Buenrostro et al. 2015; Mezger et al. 2018)
Fresh	Nuclei	Cell line, PBMC, human cortex, *Arabidopsis thaliana*, fly	(Cusanovich et al. 2015; Satpathy et al. 2019; Dorrity et al. 2021; Mich et al. 2021; Janssens et al. 2022)
Frozen	Cell	Cell line, human and mouse skin fibroblast, mouse cardiac progenitor cells, mouse splenocytes	(Chen et al. 2018b)
Frozen	Nuclei	Mouse brain, 30 adult human tissues	(Lareau et al. 2019; Zhang et al. 2021b)
Frozen	Fixed nuclei	15 human fetal tissues	(Domcke et al. 2020)

After determining the method and protocol for the scATAC-seq experiments, the next step is to ensure data quality. Regardless of the protocol followed, there are two crucial quality control steps during the experiment: sample-level quality control and library-level quality control (Fig. 1A).

To achieve sample-level quality control, first, the viability of cells or nuclei must be assessed before library construction. It is recommended that the cell viability should exceed 80%. Otherwise, the tagmentation of cell-free DNA released by dead cells might increase sequence noise and compromise data quality. In addition, accurate quantification of the cell number or nuclear concentration needs to be performed to ensure the appropriate number of captured cells. Library construction can be completed according to the detailed protocol if the sample passes the first quality control step.

For library-level quality control, it is essential to evaluate whether the chromatin landscape has been appropriately profiled prior to sequencing. This can be achieved by examining the size distribution of DNA fragments. Tn5 transposition events provide detailed information regarding nucleosome packing and positioning. As DNA molecules are protected by integer multiples of nucleosomes, the insert size distribution of sequenced fragments from chromatin exhibits an apparent periodicity of approximately 200 bp, roughly in accordance with the length of the DNA wrapped around each nucleosome. The size of the fragments in the scATAC-seq library can be examined using an Agilent Bioanalyzer. The results indicate the quality of library construction. We present in Fig. 1B, C a typical fragment distribution quantified by DNA analyzers, such as Qseq or Agilent Bioanalyzer. The peaks indicate nucleosome-free, mononucleosome, dinucleosome, and multinucleated fragments. The fragment size distribution analysis of the sequencing data also reveals a similar pattern (Fig. 1D). The comparison of fragment distribution quantified by experimental and informatics methods will help to gain empirical experience in determining the quality of a library prior to sequencing.

## General workflow for the analysis of scATAC-seq data

**Fig. 2:**
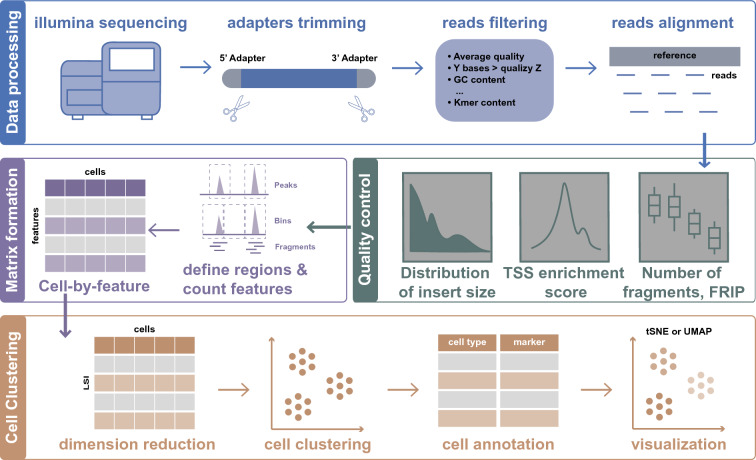
Schematic overview of scATAC-seq data preprocessing workflow

In the general processing of single-cell ATAC-seq data (including raw sequencing processing, feature-by-cell matrix formation, and dimension reduction), the primary consideration is the quality of the data library, independent of biological questions (Fig. 2). The generic workflow of raw data processing consists of the following steps. First, the adapter sequences in the raw reads are trimmed, and low-quality reads are filtered out using Trimmomatic (Bolger et al. 2014) or fastp (Chen et al. 2018a). Next, trimmed read pairs are mapped to the reference genome using tools, such as bowtie2 (Langmead and Salzberg 2012), bwa (Li and Durbin 2009), and STAR (Dobin et al. 2013). Lastly, the fragments are identified as read pairs with high mapping quality in the nuclear genome. To account for the Tn5 insertion offset, the start and end of fragments can be adjusted optionally (+4 for the plus-strand and − 5 for the minus-strand). Several raw data processing pipelines are available to researchers. The choices of packages for each step are flexible. For example, if researchers use a commercial platform such as 10X Genomics, all these steps can be easily accomplished using Cell Ranger ATAC software (https://support.10xgenomics.com/single-cell-atac).

After processing the raw sequence data, low-quality barcodes and multiples must be filtered out, by considering several cell-level quality control metrics or using model-based approaches. Three crucial metrics are commonly used for cell-level quality control. The first factor is the number of unique nuclear fragments. Cells with few fragments do not provide sufficient information to interpret, whereas those with an extremely high number of fragments may represent doublets. The other two metrics evaluate the signal-to-background ratio, including the fraction of transposition events in the peaks and the transcription start sites (TSS) enrichment scores. The idea behind these two metrics is that open chromatin regions are enriched in functional regulatory elements (peaks in ATAT-seq data), such as promoters and enhancers. A low signal-to-background ratio indicates that the chromatin structure of the cell may be disintegrated due to improper experimental manipulation. In addition to these three metrics, there are other quality control criteria. For example, the ratio of mononucleosomal to nucleosome-free fragments can be used to filter out cells without ATAT-seq-specific nucleosome banding patterns.

For each cell-level quality control metric, no single threshold is suitable for all samples. An appropriate threshold should be determined based on the characteristics of samples and species. Generally, the distributions of QC metrics are examined to determine appropriate cutoffs. Empirically, for human and mouse data, the number of unique nuclear fragments greater than 1000, the fraction of transposition events in peaks greater than 0.3, and TSS enrichment scores greater than 5 or 6 (https://www.encodeproject.org/atac-seq/) are recommended. Furthermore, thresholds of QC metrics can be adjusted after subsequent analysis, and multiplets (predominantly doublets) can be further excluded by applying advanced methods with different packages, such as AMULET (Thibodeau et al. 2021) or bap (Lareau et al. 2019).

Cells that have passed quality control are used to generate the feature count matrix, which consists of the fragment counts within each feature for each cell. The construction of the feature-by-cell count matrix can be summarized into three broad modules: defining target regions, count features, and transformation.

In contrast with the analysis of scRNA-seq data, in which genes are the target regions (where features are counted), there are various options for target regions in scATAC-seq data. In practice, researchers have the flexibility to adjust the defined target regions depending on the characteristics of the samples and the specific biological questions being addressed. With most of the current tools, such as ChromVAR (Schep et al. 2017), scABC (Zamanighomi et al. 2018), and Cicero (Pliner et al. 2018), regions are defined based on peak calling. Because per-cell scATAC-seq data are essentially binary, we cannot call peaks at the single-cell level. Peaks are usually identified using either a reference bulk ATAC-seq data or by aggregating single-cell ATAC-seq data. To avoid missing information on rare cell types, peaks can also be called and merged from pseudo-bulk data, which aggregate cells from individual clusters. In addition, there are methods such as snapATAC (Fang et al. 2021), which segments the genomes into fixed-size bins (windows) and counts the number of features within each bin.

Following the definition of the target regions, the features within these regions are counted. In most cases, the number of fragments that overlap each peak region or genome bin is counted. Some other tools, such as Cicero (Pliner et al. 2018), directly quantify the activity of each gene in the genome by summarizing the number of fragments within the gene body, the promoter region and putative distal regulatory elements.

After constructing the initial raw feature count matrix, several data transformation methods can be applied to compensate for the inherent sparsity before downstream analysis. Binarization is one of the most frequently used data transformation methods. This can alleviate potential problems arising from sequencing depth or PCR amplification artifacts. Recently, a growing number of tools have adopted latent semantic indexing (LSI) (Cusanovich et al. 2015, 2018a, b; Granja et al. 2021; Stuart et al. 2021), a natural language processing approach that was originally designed to assess document similarity based on word counts. In the case of scATAC-seq data, cells are regarded as documents, whereas peak regions are regarded as words.

After the transformation of the raw feature-by-cell matrix, dimensionality reduction is further applied to mitigate redundant features and potential noise, while preserving biologically meaningful variance, cell clustering and annotation can be performed then.

Dimension reduction techniques, such as t-distributed stochastic neighbor embedding (t-SNE) (van der Maaten and Hinton 2008), and uniform manifold approximation and projection (UMAP) (Becht et al. 2019), are used to display cells in two-dimensional space. Compared to t-SNE, which is designed to preserve the local structure of data, UMAP preserves both the local and most of the global data structure, performs faster, and better reflects the developmental chronological continuity.

To facilitate better annotation of cell types, cells with similar accessibility profiles are organized into clusters. In the scATAC-seq data, three different unsupervised clustering methods are used: K-means clustering, hierarchical clustering, and the Louvain community detection algorithm (Chen et al. 2019). The Louvain community detection algorithm was found to outperform other clustering methods in the processing of scATAC-seq data (Chen et al. 2019). After clustering, it is common to assign a cell identity to each cluster. Broadly, there are two approaches to cell identity annotation: the cell type-specific peaks-based method and the scRNA-seq-based method. Enhancers can be used to accurately annotate cell types as distal cis-regulatory elements specific to particular cell types and states. However, this method is only suitable for a limited number of datasets with known cell-type-specific enhancers. For the scRNA-seq-based method, cell type-specific gene expression is predicted based on their accessibility and used to annotate cells. In addition, scATAC-seq data can also be integrated with reference scRNA-seq data, and cell identity annotation can be transferred across the two modalities.

## Analysis of cell type-specific chromatin architecture and regulatory grammar

**Fig. 3:**
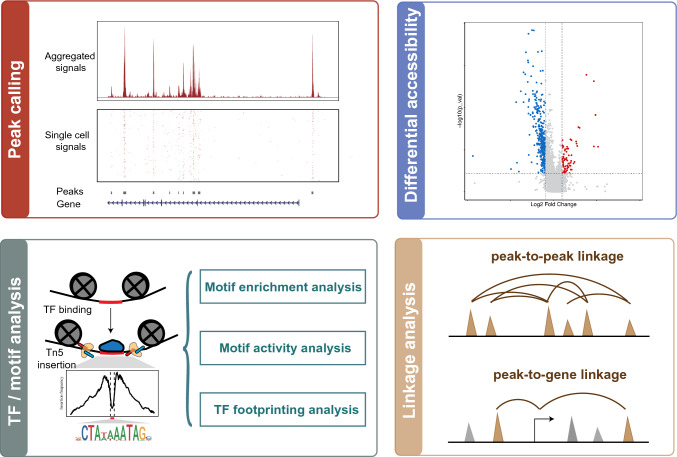
Schematic overview of epigenomic profiling from scATAC-seq data

In contrast to the general processing, which focuses on the conversion of data formats and mathematic natures of scATAC-seq data, the downstream analyses are based on the scientific hypothesis and experimental design; in other words, there is no uniform approach to this type of analysis. However, based on the chromatin accessibility profile provided by scATAC-seq, the following steps are commonly taken (Fig. 3): (1) profiling the regulatory elements for each cluster/cell type, (2) identifying differentially accessible regions between different clusters/cell types, (3) uncovering key factors that contribute to the altered chromatin accessibility, and (4) Linking promoter–enhancer interactions.

To gain insight into the cluster- or cell type-specific biology, differential accessibility analysis can be performed in two ways: cluster-specific peaks can be obtained by comparing the chromatin accessibility of cells in a particular cluster with that of all other cells. Additionally, a pairwise comparison between the two groups can be performed. A variety of statistical tests have been applied in differential accessibility analysis, including the Wilcoxon test (Yu et al. 2020), binomial test (Cusanovich et al. 2018a), Wald test (Zamanighomi et al. 2018), and logistic regression models (Stuart et al. 2021). Differential accessibility analysis may be confounded by technical biases, such as the number of unique nuclear fragments and the TSS enrichment score, which should be considered during the analysis. For example, when identifying marker peaks for each cluster, ArchR (Granja et al. 2021) selects a set of background cells that match the known biases for each cell group and performs comparisons between each cell group and its background cells. Signac (Stuart et al. 2021) uses logistic regression for differential accessibility analysis and regards the total number of fragments as a latent variable to mitigate the effects of technical biases. Differential accessibility between clusters embeds the chromatin information that regulates gene expression.

Combinations of transcription factors (TFs) orchestrate spatiotemporal genetic programs, which regulate the chromatin state and gene transcription by recognizing and binding to specific DNA sequences in cis-regulatory elements. Interpreting chromatin accessibility profiles at the single-cell level assists in deciphering key cell type-specific regulators of cellular differentiation.

Three main strategies are used to identify TFs of interest: searching for overrepresented motifs in cell type-specific accessible regions (Stuart et al. 2021), comparing motif activity between cell types (Schep et al. 2017; Stuart et al. 2021), and detecting foot-printing for TF occupancy (Li et al. 2019; Bentsen et al. 2020; Stuart et al. 2021). These three forms of analysis can be used to identify a list of candidate TFs that show considerable changes in accessibility at putative TFBSs. Because TFs from the same family can share similar motifs, they frequently show the same patterns in motif-based TF analysis, making it challenging to appropriately identify TFs of interest. To overcome this obstacle and narrow down the candidate list, scRNA-seq can be integrated to identify TFs whose gene expression is positively correlated with changes in the accessibility of their corresponding motifs. If matched gene expression data are not readily available, the gene scores of TFs predicted based on accessibility around genes can be used.

It has been shown that accessibility profiles along the linear genome in individual cells are associated with higher-order chromosome folding (Buenrostro et al. 2015). Therefore, promoter–enhancer interactions and gene regulatory networks can be obtained from scATAC-seq data, which is also known as linkage analysis. There are two primary types of linkage analysis: peak-to-peak co-accessible analysis and peak-to-gene linkage analysis. Cicero (Pliner et al. 2018) is the first algorithm developed to link distal enhancers with promoters on a genome-wide basis based on patterns of co-accessibility in scATAC-seq data. Briefly, the peak-to-peak co-accessibility analysis looks for correlations of accessibility between two peaks across cells. Thus, it does not necessarily indicate a direct regulatory relationship between inferred co-accessible peaks because cell type-specific peaks are frequently co-accessible. To overcome this challenge, peak-to-gene linkage analysis is implemented by integrating scRNA-seq data and computing correlations between peak accessibility and gene expression (Ma et al. 2020; Granja et al. 2021). Compared with peak-to-peak co-accessibility analysis, this method better reflects gene regulatory interactions.

## Genetics bonus beyond chromatin profiling from scATAC-seq

**Fig. 4:**
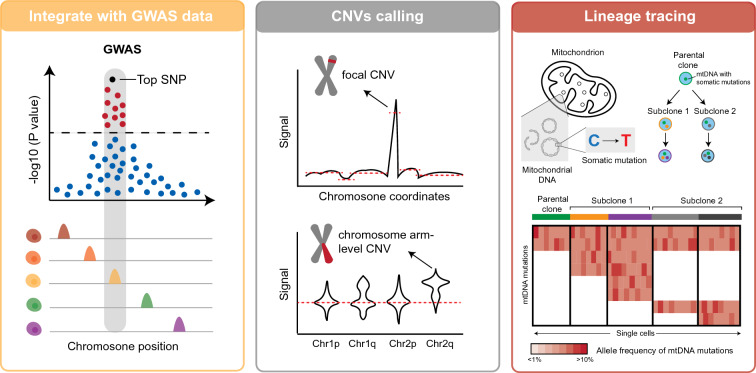
Schematic overview of genetics bonus from scATAC-seq

Although the scATAC-seq method was designed to capture the chromatin structure and epigenetic information of individual cells, as it works on DNA, the genetic information is incorporated simultaneously. The by-product genetic information from scATAC-seq data can also be used to assay whole-genome copy number variants at the single-cell level and infer cell lineage relationships based on somatic mutations on mitochondrial DNA. Furthermore, the cell type-specific cis-regulatory elements have great power to infer the function of non-coding genetic variants (Fig. 4).

The chromatin accessibility profiled by scATAC-seq provides a comprehensive map of cis-regulatory elements for many cell types at the same time. Intersecting these cis-regulatory maps with genetic variants identified by genome-wide association studies facilitates the interpretation of how non-coding genetic variants are linked to complex traits or diseases. Several studies have demonstrated a framework to systematically interpret non-coding risk variants using cis-regulatory maps (Cusanovich et al. 2018a; Rai et al. 2020; Trevino et al. 2021; Zhang et al. 2021b).

As an example, scATAC-seq from 30 adult and 15 fetal human tissue samples revealed 1.2 million cis-regulatory elements in 222 distinct cell types. Using a hypergeometric test, Zhang et al. found that GWAS variants of 450 traits/diseases were enriched in cis-regulatory elements from at least one cell type. The enrichments revealed many expected cell-type-disease phenotype relationships; for example, eczema risk variants were strongly enriched in adult T lymphocyte cis-regulatory elements, and atrial fibrillation risk variants were strongly enriched in both adult and fetal atrial and ventricular cardiomyocyte cis-regulatory elements (Zhang et al. 2021b). Besides disease-associated variants, de novo non-coding mutations in patients could also be interpreted in combination with cis-regulatory maps from scATAC-seq using deep-learning models (Trevino et al. 2021).

When ATAC-seq was applied to malignant samples and cell lines, it was found that the background signals from the ATAC-seq data could predict the karyotype and copy number variations of cells (Denny et al. 2016; Xu et al. 2017). This genetic information can also be applied to scATAC-seq to infer the copy number variations (CNVs) at the single-cell level. Several dedicated tools have been developed and used to call CNVs (e.g., focal amplifications and chromosome arm-level gains and losses) from scATAC-seq data (Ludwig et al. 2019; Nikolic et al. 2021). Copy-scAT is an R package that uses scATAC-seq data to infer copy number variants and visualize genetic heterogeneity in clinical samples (Nikolic et al. 2021). It takes barcode-fragment matrices generated by Cell Ranger ATAC as input to create a pileup of total coverage over bins of 1 Mb. Subsequently, large peaks in the normalized coverage matrices are used to infer focal CNVs. In a recent study (Nikolic et al. 2021), Copy-scAT was shown to be effective in detecting CNVs of diverse malignancies at the single-cell level using scATAC-seq data. The authors found that malignant and non-malignant cells could be distinguished based on CNV status (the presence or absence of CNVs). It was also determined that cells sharing a specific CNV tend to cluster together in the scATAC-seq data, which suggests that genetics may contribute to a particular epigenetic profile.

Mitochondrial DNA is amplified simultaneously when scATAC-seq is performed on whole cells instead of nuclei. Cell-specific somatic mutations in the mitochondrial genome can be efficiently detected simultaneously. Several studies have shown that it is feasible to utilize this endogenous genetic information to trace cell lineages in various human cell types (Ludwig et al. 2019; Xu et al. 2019). Furthermore, an optimized scATAC-seq protocol based on the 10X Genomics platform has been developed to combine mitochondrial genotyping and chromatin profiling on a large scale (Lareau et al. 2021). The genetic information on the mitochondrial genome is generally processed separately using an SNP calling pipeline, such as GATK (McKenna et al. 2010). In brief, the mitochondrial sequences should be included in the reference genome during alignment. Reads mapped to the mitochondrial genome with high confidence are extracted and de-duplicated. Next, the reads are realigned to correct potential mapping errors, thus enabling accurate SNP calling. Following this, variant calling tools, such as VarScan2 (Koboldt et al. 2012), can be applied to call somatic mutations in the mitochondrial genome at the single-cell level. High-confidence mutations are retained based on several quality control metrics, including sequencing coverage and strand balance. The variant allele frequency (VAF) for each mutation in each cell is computed, thereby removing germline mutations with a high VAF and constructing a cell-by-variant matrix. Lastly, the matrix can be used to construct lineage relationships among cells using traditional phylogenetic methods or advanced methods (Lin et al. 2022).

## Key analytical tools for scATAC-seq data

With the rapid development of scATAC-seq technologies, a growing number of packages have been developed to analyze scATAC-seq data. These packages can be divided into two categories: tools that use raw sequence data as input for general data processing and those that use processed data as input for various downstream analyses.

Cell Ranger ATAC is one of the most popular tools used for primary processing and initial downstream analysis (e.g., identifying open chromatin regions, motif annotations, and differential accessibility analysis). Despite its convenience, it requires extensive computational resources and lengthy runtime. To address this, a pseudo-alignment approach was introduced into the scATAC-seq preprocessing pipeline with negligible loss in accuracy (Cittaro et al. 2020). Furthermore, the alignment algorithm can also be improved to accelerate the pipeline (Zhang et al. 2021a). However, a significant drawback of one-stop pipelines is their inflexibility in selecting the methods and parameters for specific analysis tasks.

Instead of one-stop pipelines, numerous packages use preliminarily processed data as input for various downstream analyses. Several packages provide comprehensive analysis frameworks that cover virtually all aspects of the previously mentioned downstream analyses, such as Signac (Stuart et al. 2021), ArchR (Granja et al. 2021), SnapATAC (Fang et al. 2021), scATAC-pro (Yu et al. 2020), and APEC (Li et al. 2020). In Table 2, we summarize the capabilities of the downstream analysis for these packages. The inherent sparsity of scATAC-seq data presents methodological challenges. Therefore, instead of offering a complete pipeline, several packages have been developed to improve a particular aspect of analysis. For example, AtacWorks (Lal et al. 2021) and SCATE (Ji et al. 2020) enhance scATAC-seq signals, thereby facilitating the investigation of regulatory elements in rare cell subpopulations.

**Table 2:** Summary of the features supported by five recent scATAC-seq software packages that provide comprehensive analysis frameworks

	Signac	ArchR	SnapATAC/ SnapATAC2	scATAC-pro	APEC
Language	R	R	R/Python	Shell, R	Python
Input files	Fragment files, Peak-by-cell matrix	Fragment files/BAM files	Fastq files Snap files	Fastq files/Fragment files/BAM files	Fastq files/Peak-by-cell matrix
Quality control	✓	✓	✓	✓	✓
Doublet removal	✗	✓	✓	✗	✗
Feature matrix	Peak	Bin, peak	Bin, peak	Peak	Peak
Data imputation	✗	✓	✓	✗	✗
Gene activity	✓	✓	✓	✗	✓
DR, clustering	✓	✓	✓	✓	✓
Peak calling	✓	✓	✓	✓	✓
DAR	✓	✓	✓	✓	✓
Functional annotation	✗	✗	✓	✗	✓
Motif enrichment	✓	✓	✓	✓	✓
Motif activity	✓	✓	✓	✓	✓
TF foot-printing	✓	✓	✗	✓	✗
Peak-to-peak	✓	✓	✗	✓	✓
Peak-to-gene	✓	✓	✓	✗	✗
Trajectory	✓	✓	✗	✗	✓
Genome browser	✓	✓	✓	✓	✓
Batch effect correction	✓	✓	✓	✗	✗
scRNA integration	✓	✓	✓	✗	✓
Reference	(Stuart et al. 2021)	(Granja et al. 2021)	(Fang et al. 2021)	(Fang et al. 2021)	(Li et al. 2020)

Although various software packages are available for scATAC-seq analysis, there is no consensus on which is the best. We recommend that beginners start with one-stop pipelines for general data processing and then move on to packages with comprehensive analysis frameworks and detailed tutorials, such as Signac (Stuart et al. 2021), ArchR (Granja et al. 2021), and SnapATAC (Fang et al. 2021), to gain a better understanding of scATAC-seq analysis. Recently, ten computational methods for scATAC-seq data analysis were compared, with the result that SnapATAC (Fang et al. 2021), Cusanovich2018 (Cusanovich et al. 2015, 2018a, b), and cisTopic (Bravo González-Blas et al. 2019) were found to outperform other methods (Chen et al. 2019). With the ongoing development of the scATAC-seq analysis tools, more in-depth benchmarking studies are required.

## Prospects

Although both experimental and computational methods have been developed for mammalian cells, the application of scATAC-seq is not restricted to mammalian species. Based on the principle of scATAC-seq, its application to plants is straightforward as long as the nuclei can be accessed from the target samples. Successful protocols for performing scATAC-seq have been reported in the case of maize (Marand et al. 2021) and *Arabidopsis thaliana* roots (Dorrity et al. 2021). These protocols can be readily adapted to other plants and extended to different organs. The epigenetic insights provided by scATAC-seq will benefit basic and applied research in fields as diverse as biomedicine and agricultural science.

## Data Availability

Data sharing not applicable to this article as no datasets were generated or analyzed during the current study.
